# Lifestyle Factors and Their Influence on Rheumatoid Arthritis: A Narrative Review

**DOI:** 10.3390/jcm11237179

**Published:** 2022-12-02

**Authors:** Christoph Schäfer, Gernot Keyßer

**Affiliations:** Department of Internal Medicine II, Medical Faculty, Martin Luther University Halle-Wittenberg, 06120 Halle, Germany

**Keywords:** rheumatoid arthritis, lifestyle, diet, Mediterranean diet, smoking, socioeconomic status, physical activity

## Abstract

In recent years, a possible association of lifestyle factors with rheumatoid arthritis (RA) has attracted increasing public interest. The aim of this review is to provide an overview of the extent and the limitations of current evidence regarding lifestyle factors and RA. The PubMed medical database was screened for epidemiological and prospective studies investigating the contribution of lifestyle factors to the development and the course of the disease. Large epidemiological studies have identified smoking, unhealthy diet and adiposity, as well as a low educational level and low socioeconomic status, as factors that increase the incidence of RA. In addition, several lifestyle habits influence the response of RA to antirheumatic drugs. Among others, smoking, obesity and poor physical activity are associated with a worse treatment outcome. Methodological problems often impair firm conclusions with respect to the causal role of these factors in the risk and the course of RA. However, current evidence is sufficient to recommend a healthy diet, the prevention of obesity, the cessation of smoking and the maintenance of a high level of physical activity to support the effectivity of modern antirheumatic medication.

## 1. Introduction

Patients with inflammatory rheumatic diseases (IRDs) often express their desire to ensure the success of medical treatment or to replace antirheumatic drugs, at least partially, by changing lifestyle factors. This desire is fostered by the plethora of media reports that describe the influence of nutrition, body weight, physical fitness and stress level on health and life expectancy [1]. In addition, patients with rheumatic diseases represent a target audience for advertisement campaigns soliciting food items with real or putative health-promoting features and for other commercial offers that promise improved fitness, a healthier life and an increased level of well-being [2,3].

However, under the scrutiny of evidence-based medicine, only a handful of lifestyle factors are of proven influence for patients with IRD. Most data in this respect are provided for rheumatoid arthritis (RA), clearly showing that lifestyle is important with respect to the risk of this disease. Recently, the Nurses Health Study (NHS) cohorts were analyzed with respect to the impact of modifiable lifestyle factors (cigarette smoking, being obese, lacking physical activity and consuming unhealthy diets, as well as drinking alcohol in excess) on the risk of RA. A healthy lifestyle index score (HLIS) was developed to quantify these five factors [4]. In general, a higher (healthier) HLIS was associated with a lower hazard ratio (HR) for RA, most prominently in women with five healthy lifestyle factors (HR 0.42), which was even lower for seropositive RA (HR 0.24) [4].

The aim of this review is to provide an overview of the extent and the limitations of current evidence regarding lifestyle factors and RA.

## 2. Nutritional Factors for the Etiology and the Course of RA

The question as to whether nutritional factors contribute to the pathogenesis of RA is difficult to answer. Human food consists of variegated components. Its origin, preparation and quality is highly variable and changing with time. However, it is now well-established that food can influence the inflammatory milieu of the human body [5] via several mechanisms. Nutritional habits determine the composition of our microbiome, which is increasingly recognized as a major player in the etiology of IRD [6]. In a non-RA cohort, a diet with high fiber content resulted in a reduced level of inflammatory cytokines [7]. In addition, polyunsaturated fatty acids may exert anti-inflammatory and immunomodulatory effects, as illustrated by the inverse correlation between n-3-fatty acids and autoantibodies in individuals at high risk for RA [8].

However, it takes large databases to demonstrate that these factors are of real importance with respect to the risk of IRD. One such database is the Sister Study Cohort of the National Institute of Environmental Health Sciences (NIEHS) [9]. By analyzing the data of more than 50,000 women, nutritional factors such as low birthweight and poor nutritional status in childhood were identified as contributors to RA. The study did not identify specific food items to account for RA pathogenesis. This was done in more detail by the NHS I and II studies, in which more than 1000 cases of newly diagnosed RA occurred in a study population of more than 150,000 women [10]. Participants had to fill in food frequency questionnaires biennially, thereby providing the basis to calculate the alternative healthy eating index (AHEI 2010). A high score indicates a diet that is rich in fruits, vegetables, nuts, highly unsaturated fatty acids and whole-grain products, including moderate alcohol consumption. The score decreases with the consumption of red or processed meat, soft drinks, sodium and saturated fatty acids. Among women younger than 55 years, a high AHEI 2010 was associated with a hazard ratio for RA of 0.67 compared with patients with the lowest AHEI 2010 [10]. Subanalyses of the NHS data revealed that a higher alcohol score (moderate intake) or a lower intake of red meat was significantly associated with a reduced RA risk, which was independent of the current body mass index (BMI) and other covariates [10]. The regular consumption of sugar-sweetened soda alone also led to an increased risk for RA in the NHS cohort [10].

By correlating the intake of 39 predefined food items with the levels of inflammatory markers measured in the NHS, the Empirical Dietary Inflammatory Index (EDII) was developed in a non-RA cohort to identify a dietary pattern most predictive of increased values of interleukin 6, C-reactive protein (CRP) and tumor necrosis factor alpha receptor 2 [11]. Components positively associated with inflammatory markers include, among others, red and processed meat, refined grains and high-energy beverages, whereas wine, coffee and leafy green vegetables were found to be negatively associated with inflammatory markets [11]. A higher EDII was associated with an increased RA-risk [12]. Recently, these results were confirmed by a large cross-sectional study of Korean patients, showing a significantly lower prevalence for RA, as well as osteoarthritis and cardiovascular disease, in a cohort with a higher-than-average consumption of n-3-fatty acids, fruits and green vegetables [13].

The NHS data could not demonstrate a connection between the adherence to a Mediterranean diet (MD) and the risk of developing RA [14]. The Mediterranean diet is characterized by a high intake of legumes and fruits, with olive oil as the main source of lipids, as well as a reduced share of red meat, high-fat dairy products and refined sugar. However, a population-based case control study, the Swedish epidemiological investigation of RA (EIRA) study, demonstrated a significant risk reduction for seropositive RA of 21% in persons with a strong adherence to MD, although this was observed in male participants only [15].

### 2.1. Fish and Polyunsaturated Fatty Acids (PUFA)

Polyunsaturated oils such as fish oil and various plant oils exert anti-inflammatory and antiarteriosclerotic effects. Several studies have reported a trend towards a protective role of fish, especially oily fish species, although the observed effects did not achieve statistical significance [16,17,18]. The analysis of more than half a million subjects registered in the British Biobank demonstrated that the consumption of oily fish, breakfast cereals and a moderate alcohol intake was negatively associated with the risk of RA [19]. In addition, a dose-dependent effect of the intake of polyunsaturated fatty acids (PUFA) was observed among 32,232 elderly women, showing that a long-term intake of more than 0.21 g/d was associated with a risk reduction for RA of 52% [20]. In a cohort of pre-RA patients, the erythrocyte levels of n-6-PUFA were inversely correlated with the risk of RA, with an odds ratio (OR) for definite RA of 0.29 for patients with the highest tertile of PUFA compared with the lowest [21]. The content of n-3-PUFA in erythrocytes is also inversely correlated with the prevalence of rheumatoid factor (RF) and cyclic citrullinated peptide (CCP) antibodies in sera of patients genetically at risk for RA [8].

### 2.2. Adiposity and the Risk for RA

The effect of a high EDII on RA risk mentioned above was attenuated after adjusting for BMI [12]. In addition, a recent analysis of the NHS cohort revealed that obesity had the most marked effect on RA risk [4], and after adjustment for the BMI, the AHEI 2010 did not contribute independently to the hazard ratio for RA [4]. By analyzing the data of 108,000 women who were followed-up prospectively between 1989 and 2017, the NHS demonstrated that weight gain from baseline was quantitatively associated with the risk for seropositive and seronegative RA. Women gaining more than 20 kg had a relative risk (RR) of 3.8 for seropositive RA compared with women who maintained a stable weight [22]. Therefore, this study elegantly confirms previous data that suggested a connection between the BMI and the risk of RA, albeit with considerable heterogeneity [23]. Significantly, the combination of adiposity and smoking was disproportionally associated with the development of definite RA in a preselected cohort of persons without arthritis but positive for RA-associated autoantibodies compared with each component alone [24].

It was hypothesized that central adiposity may be of prominent importance for an elevated RA risk. A British cohort analysis revealed that central adiposity, assessed by waist circumference, was associated with an elevated odds ratio for the prevalence of RA and psoriatic arthritis. This association was stable, even after adjustment for confounders and BMI [25]. However, these data were collected in a sample with established RA. A prospective study of the NHS cohort did not reveal an independent contribution of abdominal adiposity in comparison to general adiposity [26].

### 2.3. Alcohol

It is now well accepted that low-to-moderate alcohol consumption is a protective factor with respect to RA (review in [27]). According to data from 30,447 participants of the Malmö Diet and Cancer Study, persons with moderate baseline alcohol consumption (3.5–15.2 g/day vs. <3.5 g/day) tended to have a reduced risk of RA (OR 0.48) compared to participants who drank less than 3.5 g alcohol per day. The study was adjusted for smoking and the level of education [28]. A meta-analysis of eight prospective studies involving more than 195,000 participants concluded that low-to-moderate alcohol consumption lowered the risk for RA to a RR of 0.86 compared with the risk of abstinent persons. The same analysis described a non-linear relationship between alcohol consumption and RA- risk. The protective effect was largest with an average daily consumption of 9 g alcohol per day, compared with 3 g or 12 g per day. The effect waned and even trended towards a negative influence with doses reaching 30 g alcohol per day [29]. The type of the preferred alcoholic beverage was of no significance [29].

### 2.4. Nutritional Intervention for the Treatment of RA

Nutritional studies to investigate the impact of food on the activity of IRD are difficult to perform. A blinding of treatment arms is not possible, and the risk of selection bias is high. In 2009, a Cochrane analysis of 14 clinical trials including 837 patients concluded, “The effects of dietary manipulation… on rheumatoid arthritis are still uncertain due to the included studies being small, single trials with moderate to high risk of bias” ([30], page 2). Recent systematic reviews of the literature came to the same conclusion, i.e., the role and the efficacy of dietary interventions are not clearly defined, and heterogeneity and bias remain significant problems [31]. The evidence for any dietary intervention in RA has to be graded as low or very low, primarily owing to the limited number of studies with small sample sizes [32]. The main impact of dietary interventions on RA, if any, is pain reduction rather than objective measures of disease activity [33].

In general, dietary interventions are not suitable to replace effective disease-modifying antirheumatic drugs (DMARDs). In addition, no data demonstrate the ability of diet to either prevent structural damage or to be effective against highly active disease. Significantly, a far-reaching interference into accustomed dietary habits may be difficult to tolerate for patients and may lead to early discontinuation of clinical studies [34]. Therefore, the recently published recommendations of the European League Against Rheumatism (EULAR) regarding lifestyle behaviors for IRD patients are very cautious in recommending any specific diet besides a “healthy balanced diet” and a “healthy weight” [35]. Nevertheless, some studies make the cautious conclusion that Mediterranean and anti-inflammatory diets may be recommended for patients with IRD. A Mediterranean diet leads to reduced levels of inflammatory cytokines in RA patients compared with controls [36]. In addition, there is now evidence that diets rich in anti-inflammatory components such as olive oil, nuts or fatty fish reduce cardiovascular risk [37,38,39]. Although these data were generated in non-RA samples, we assume that this finding is of major importance for patients with inflammatory arthritis, given that these patients have significantly elevated risk for major cardiovascular events [40].

One of the earliest studies that investigated the role of nutrition in RA was a randomized controlled trial that combined a fasting period of approximately one week with three to five months of a vegan diet, followed by lactovegetarian nutrition. The study demonstrated small but significant improvements in swollen joint count and patient global assessment, which were mainly achieved during the fasting and maintained thereafter. However, 40 of 53 participants in the study did not receive DMARD treatment. Therefore, the disease activity of the sample was probably very low [41].

In 2003, a randomized controlled trial investigated the impact of an MD on RA disease activity over 12 weeks. The intervention resulted in a small albeit significant improvement in the Disease Activity Score (DAS28) [42]. The effect correlated with the extent of the uptake of n-3-PUFA [43] and was not dependent on the weight reduction that occurred during this and other dietary interventions [44]. More recently, in a randomized cross-over study, the anti-inflammatory diet in RA (ADIRA) trial, the participating RA patients consumed a diet designed to exert anti-inflammatory effects. The diet was rich in legumes, fruits and fish and was supplemented with probiotics [45]. The primary endpoint was not met, probably because the study was underpowered. However, the DAS28 improved in an unadjusted analysis.

Taking these studies together, it can only be speculated whether the association of inflammatory markers with nutrition observed healthy subjects [7,11] translates into reduced disease activity in RA patients. Although [42,45] both point in this direction, definitive evidence supporting this hypothesis is still pending.

### 2.5. Fasting and the Activity of RA

Besides the study mentioned above that included a fasting period [41], a meta-analysis of this and three other clinical trials argued for pain reduction after controlled fasting not exceeding 12 weeks [46]. However, there is neither evidence for long-term effects, especially with respect to structural damage, nor for a benefit of repeated fasting.

### 2.6. Exclusion Studies in RA

In recent decades, the possibility of food allergens contributing to the pathogenesis of RA was discussed intensely. However, a study eliminating milk allergens and azo dyes in a double-blinded fashion did not convincingly demonstrate a clinical effect in RA [47], although these phenomena could be relevant for subgroups of patients. In a 2001 randomized study, RA patients were put on a vegan, gluten free diet. The intervention resulted in an improvement in the American College of Rheumatology response criteria (ACR20) and a decrease in CRP levels. However, more than 40% of the study population terminated the trial ahead of schedule, indicating the low tolerability of this rather radical dietary regimen [34]. A small study on obese RA patients with stable disease revealed that weight reduction resulted in additional benefit if the balanced diet was deprived of meat, gluten, lactose and all dairy products, compared with a balanced diet alone. Patients consuming the privative diet achieved reduced blood pressure, as well as improvements in physical and mental health, compared with the control group [48]. However, potential drawbacks of these exclusion diets, namely a lack of dietary fiber, calcium or essential amino acids, outweigh the potential advantages. Therefore, the exclusion of gluten (for non-celiac patients) and dairy products is explicitly not recommended for patients with IRD [49].

### 2.7. Interventions Aiming at Weight Reduction in RA

A retrospective analysis of RA patients who underwent bariatric surgery demonstrated a marked reduction in inflammatory markers, as well as reduced RA disease activity after the surgical intervention [50]. In a randomized study, RA patients were put on a hypocaloric diet of 1000–1500 kcal/day for 12 weeks. Besides a weight loss of 9.5 kg, these patients experienced significant improvements in disease activity (DAS28) and the health assessment questionnaire (HAQ) disability index [51].

### 2.8. Nutritional Supplements

Reports arguing for beneficial effects of nutritional supplements for IRD have been published in large numbers. In a broader sense, some components of daily food may be considered a supplement, namely spices such as curcumin, cinnamon, garlic and saffron. These food ingredients possess multiple anti-inflammatory and antioxidative features in vitro and in experimental models [52,53]. However, interventional studies applying these compounds in RA are of poor quality and have a high risk of bias [52].

Probiotics contain living microorganisms such as lactobacilli or yeast. Several smaller studies have claimed a symptom-modifying effect of probiotics in IRD [54]. However, in general, these studies are highly heterogeneous and include small patient groups with low disease activity [55,56,57]. Therefore, the application of probiotics is not recommended for the adjuvant treatment of RA [58].

Of all nutritional supplements, omega-3 PUFAs have been studied most extensively in RA patients [59]. In a Cochrane meta-analysis, omega-3-PUFAs were found to significantly reduce patient-reported joint pain intensity, morning stiffness and non-steroidal anti-inflammatory drug (NSAID) consumption [59].

Experimental studies argue for a pathogenetic role of trace elements such as zinc and cadmium in RA [60], and deficiencies of zinc, copper, magnesium and selenium have been described in the sera or in the diet of RA patients [60,61,62,63]. However, there is no convincing evidence that the supplementation of either trace elements alone or a mixture of micronutrients is of any benefit with respect to disease activity in RA [64,65,66].

Vitamin E was formerly regarded as an antioxidant with anti-inflammatory potential. However, vitamin E supplementation was not associated with a reduced risk of RA development in the Women’s Health Study [67], and an excessive consumption was even linked to an increase in all-cause mortality [68]. Although a recent meta-analysis argued for the beneficial effect vitamin E in RA [69], the dietary recommendations of the French Society for Rheumatology explicitly advise against the supplementation of vitamins, including vitamin E or trace elements [49].

It is common sense that a relevant deficiency of vitamin D and iron has to be treated in RA patients. Beyond that, however, an over-supplementation is of no use and should be avoided [49].

A brief overview of studies on the influence of nutrition is provided in Table S1 in the Supplementary Materials.

### 2.9. Nutrition and Rheumatoid Arthritis: Current Recommendations

With respect to the literature presented in this review, it would be beyond the scope of the article to derive firm recommendations for patients with established RA or for persons at risk of developing this disease. However, recent efforts have been made to develop nutritional recommendations for RA patients on the basis of the current scientific evidence. The European League Against Rheumatism (EULAR) recommends a healthy balanced diet and a healthy weight for patients with rheumatic and musculoskeletal disease [35]. Recently, the French Society for Rheumatology formulated nutritional recommendations for RA patients in more detail [49], including eight general principles and nine recommendations. The general principles stress the importance of a healthy diet as one component of the overall care. The importance of nutrition with respect to cardiovascular risk is addressed, as well as the consideration of the cultural and socioeconomic background of the patient. Recommendations include a reduction in overweight, a Mediterranean diet and supplementation with polyunsaturated fatty acids. Explicitly, the recommendations do not include supplementation with vitamins, trace elements or probiotics, nor the application of a vegan or gluten-free diet. Furthermore, the elimination of dairy products is not advised [49].

## 3. Smoking and Development of RA

Exposure to tobacco smoke is the major known environmental risk factor for developing RA [70] and seems to be attributable to 20% of all RA cases and 35% of anti-citrulline antibody (ACPA)-positive RA cases [71]. A long-suspected causal relationship between smoking and the development of RA was confirmed by a twin study in 1996 [68]. Here, 79 monozygotic and 71 dizygotic twin pairs, with one twin of each pair suffering from RA, were examined for their smoking habits. In twin pairs discordant for smoking habits, smoking increased the risk of developing RA by a factor of four [72]. This effect was even more pronounced in genetically identical monozygotic twin pairs (OR: 5.5).

A deeper insight into the apparent interaction of genetic factors and smoking in the development of RA is provided by a Swedish case–control study of 858 incident RA cases and 1048 controls. This study showed a strong association between shared epitope (SE), smoking and the development of seropositive RA [73]. The SE gene resulted in an RR of RF-positive RA of 2.8 in non-smokers. In current smokers without SE genes, RR was 2.4, and in current smokers with SE genes, RR was 7.5. The highest RR of 15.7 was observed in current smokers carrying double SE genes. The risk of developing seronegative RA was not influenced by the SE gene, smoking or a combination of the two [73]. This study was confirmed by recent data [74]; in a Swedish cohort (Epidemiological Investigation of RA (EIRA)), smoking was associated with the occurrence of ACPA in a dose-dependent manner. Furthermore, the risk of developing ACPA-positive RA under the influence of smoking was strongly dependent on the presence of SE. Whereas smoking increased the risk of developing ACPA-positive RA by only 1.5-fold in the case of SE negativity, the RR increased by 6.5-fold in the presence of a single SE copy and by 21-fold in the presence of two SE copies. There was no association between SE, smoking and the development of ACPA-negative RA [74].

Smoking leads to lung disease and periodontitis and thus to chronically increased inflammatory activity. Furthermore, nicotine can drive autoimmunity via multiple pathways. Smoking activates peptidyl-arginine deaminase in lung tissue, leading to increased citrullination of proteins [75,76]. Subsequent ACPA formation predisposes the HLA-DRB1-positive population to the development of seropositive RA [73]. Independently, nicotine induces neutrophil extracellular trap (NET) formation and may also lead to ACPA formation via this pathway [77]. In an animal RA model, nicotine administration led to increased NETosis and enhanced disease activity. These experimental results suggest that nicotine could possibly trigger RA, even without fine dust inhalation, e.g., in the form of e-cigarettes [77].

The evaluation of a large cohort of 6239 Japanese RA patients showed a clear positive correlation between nicotine consumption and the occurrence of ACPA and RF [78]. Moreover, RF formation was more dependent on nicotine consumption than ACPA formation. Whereas nicotine consumption caused ACPA development only in patients with the SE, RF was induced by nicotine independently of SE. An increased ACPA level was still detected 20 years after cessation of nicotine use [78].

### Smoking and Outcome of RA

In patients with RA, smoking is associated with a poorer prognosis of the disease. A poorer response to conventional synthetic DMARDs and TNF alpha inhibitors has been demonstrated in smokers [79,80]. However, in retrospective studies, cessation of nicotine use after the onset of RA no longer appeared to favorably influence disease progression [81,82]. Passive nicotine exposure of non-smokers also had no effect on disease activity in the Swedish BARFOT study, with a very high burden of passive smoking in this cohort, affecting 68% of non-smokers [83], which is markedly higher than the rate of passive smokers in the general population, implying a role of this factor in RA pathogenesis.

Smoking is also of relevance with respect to the comorbidities associated with RA. Among them, interstitial lung disease (ILD) is found in up to 10% of patients with RA [84,85]. Both high RF and ACPA titers, as well as smoking, are associated with the occurrence of ILD [86,87]. Nicotine-induced citrullination of peptides seems to be a relevant factor for the development of ILD [76].

## 4. Fine Particulate Matter

Another environmental factor that fosters the development of both ILD and RA is exposure to particulate matter. There are numerous reports of an increased risk of RA due to inhalation exposure to silica among coal miners. The simultaneous occurrence of pulmonary fibrosis and rheumatoid arthritis in miners has become known as Caplan’s syndrome [88]. In the Swedish EIRA study, men with increased exposure to stone dust had a twofold increased risk of developing RA [89]. In a later analysis of a larger cohort, silica exposure significantly increased the risk of ACPA-positive RA, with an OR of 1.7. ACPA-negative RA did not occur more frequently after silica exposure. An excessively increased risk of developing ACPA-positive RA was observed in current smokers with silica exposure (OR 7.4) [90]. In contrast to the risk from silica exposure, the data from the EIRA study and NHS study did not show an association between estimated general exposure to fine particulate matter and the occurrence of RA [91,92]. However, in a nationwide Italian retrospective observational study of 81,363 subjects, a concentration-dependent association was detected between measured fine particulate matter exposure and the occurrence of autoimmune diseases [93]. The risk of developing RA was increased by exposure to particulate matter with a particle size of 10 µm or less and, even more significantly, to a particle size of 2.5 µm or less.

In summary, the data presented on smoking, silica dust and fine particulate matter exposure suggest an association between inflammatory processes in lung tissue and the development of ACPA-positive RA.

## 5. Socioeconomic Status

A correlation between socioeconomic status and health is common knowledge. In general, high socioeconomic status seems to be associated with better health and vice versa [94]. Socioeconomic status is associated with many individual factors that also influence the course of the disease and the development of RA, including, in addition to smoking habits, nutrition, obesity, marital status, etc. Nevertheless, several observational studies argue for the dependence of disease activity or functional impairment of RA on socioeconomic status as an independent factor.

An English cohort study of 869 consecutive RA patients showed a correlation between lower socioeconomic status and lower level of education and a higher disease activity at baseline and during the course of the disease. Increased disease activity was mainly shown with respect to functional parameters such as the HAQ, grip strength or joint score but was not reflected in the radiological course or the erythrocyte sedimentation rate (ESR) [95]. The formal level of education is often considered a surrogate parameter for socioeconomic status. In an American study of 385 RA patients, those with lower levels of education showed increased disease activity and functional limitation as measured by ESR, grip strength and joint count [96]. A total of 814 RA patients prospectively enrolled in treatment studies in England and Scotland were examined for a correlation between disease activity and social deprivation. Patients from socially deprived areas showed more impaired joint function as measured by the HAQ but no significant differences in laboratory values [97]. A recent literature review analyzed 30 studies investigating the association between social status and disease activity in RA. Among the reviewed studies, 25 reported a clear association between low socioeconomic status and increased disease activity. However, the comparability of these studies is limited by varied definitions of socioeconomic status and disease activity [98].

Whereas a dependence of the course of the disease on socioeconomic factors seems to be well established, data regarding social status as a risk factor for the development of RA are scarce. In the Swedish case–control study EIRA, the association of the development of RA with socioeconomic status, as measured by education and occupation, was investigated in 930 incipient RA cases and 1126 controls. After adjustment for age, residential area and smoking, there was still an increased relative risk of 1.4 for the occurrence of RA in the group without a university degree. This effect was pronounced for RF-positive RA (RR 1.6). Similar effects were observed after the stratification by occupation; high-ranking, non-manual employees showed a 20% lower risk of developing RA compared to other non-manual employees [99].

## 6. Psychosocial Stress

Chronic psychosocial stress is discussed as another possible factor influencing the development of chronic diseases, and patients often suspect psychological stress as the trigger of their disorder. Psychoneuroendocrinological theories postulate that chronic psychosocial stress influences the immune system via the corticotropic axis and can lead to chronic diseases [100,101,102]. A recent review on psychological stress and RA distinguishes between role stress, social stress and work stress [103]. The 16 studies analyzed revealed a considerable heterogeneity in measurement tools and definitions of psychological stress. In general, work stress and social stress were more pronounced in patients with RA than in healthy control populations. Whereas the influence of pain and chronic illness on mental health and psychological stress is well documented [103,104], there is limited literature on the possible influence of psychological stress on the development of RA.

Data from the Swedish EIRA study were examined for the influence of psychosocial stress at work on the development of RA [105]. RA cases and controls were classified by means of self-assessment and according to a job exposure matrix with regard to psychological demand and decision latitude in the job. Low decision latitude was significantly associated with an increased risk of developing RA (OR 1.6). High psychological job demands showed a trend towards a reduced risk of developing RA. These effects were still demonstrated after adjustment for the social class [105]. A Danish population-based survey of 19,890 participants reported an association between loneliness and the prevalence of RA (OR 1.3). However, owing to the design of the study, it is not possible to draw any conclusions with respect to causality [106].

The prognosis of established RA also seems to be influenced by psychosocial stress. In the randomized controlled CareRA-trial, patients with early arthritis were more likely to lose the state of remission if they were assigned to a group with a high psychosocial burden [107].

## 7. Marital and Family Status

Patients with RA are no more often divorced than comparable persons [108]. Significantly, married patients experience a lower rate of progression of disability [109]. However, only patients living in a non-distressed marriage had less pain and physical disability in comparison to unmarried patients [110]. Both results argue for the value of social support and security in coping with IRD.

Sexual dysfunction occurs more frequently in RA patients compared with the healthy population, although this factor is rarely discussed with the treating rheumatologist [111]. The problem is aggravated by pain and by the number of comorbidities [112] and associated with depression and fatigue [111]. In addition, the disease activity, the age of patients and partners and poor sleep quality contribute to problems with sexual life that occur in almost half of female RA patients [113].

There are currently no data available with respect to the influence of parenthood on RA disease activity or outcome. In contrast, a recent investigation of 23,981 parent–child pairs in whom at least one of the parents had RA reported an increased rate of mental disorders in children born to a mother with RA. There was a higher risk for autism spectrum disorders, attention-deficit/hyperactivity disorder, bipolar and major depressive disorder [114].

## 8. Physical Activity

There is little doubt about the beneficial effects of regular physical activity on factors such as quality of life, cardiovascular fitness and muscle strength in patients with RA [115]. Accordingly, recommendations have been made by EULAR for regular physical exercise for this patient group [116]. However, it remains unclear whether physical exercises can also improve the inflammatory disease activity of an existing RA. A meta-analysis that included studies on ankylosing spondylitis and systemic lupus erythematosus, as well as studies on RA, demonstrated a decrease in inflammatory activity through physical exercise across all disease groups [117]. In contrast, two meta-analyses examining the effects of cardiovascular or resistance exercises in RA studies found no effect on inflammatory activity as measured by the DAS28 [118,119].

A single study described damage progression of large joints in patients with pre-existing extensive damage as a result of high-intensity weight-bearing exercise [120]. However, all studies and meta-analyses consider even resistance exercise to be safe and beneficial in patients with RA [115,117,119,121]. Therefore, EULAR recommends not only aerobic exercise but also explicit muscle strength exercises for patients with RA [116].

Data on the possible protective effect of physical activity on the development of RA are inconsistent. In the prospective observational Nurses’ Health Study II of 113,366 women with 506 incident RA cases, recreational physical activity was dose-dependently associated with a reduced risk of developing RA. Recreational physical activity of 4 < 7 h/week and ≥7 h/week was associated with a reduced risk of developing RA (RR 0.84 and RR 0.67) compared to <1 h/week [122]. The Swedish Mammography Cohort included 30,112 women aged 54 to 89 years with 201 incident RA cases. In this study, women in the highest category of physical activity (more than 20 min per day of walking/bicycling and more than 1 h per week of exercise) had a statistically significant lower risk of developing RA compared with women in the lowest category (RR 0.7) [123]. The cohort of the prospective Iowa Women’s Health Study consisted of 31,336 women aged 55 to 69 years, of which 158 developed RA during the observation period. The level of leisure exercises stratified into low, medium and high did not correlate with the development of RA in this study [124]. A meta-analysis including four studies involving 255,365 women with 4213 incident RA cases found a negative association between physical activity and the development of RA, with a relative risk of 0.8 for the highest vs. lowest physical activity. However, a Mendelian randomization by the same authors suggested no causal relationship between physical activity and the occurrence of RA [123]. In addition to a possibly inadequate statistical power of the Mendelian randomization, this could also indicate insufficient adjustment for confounders in the studies included in the meta-analysis.

Difficulties in adjusting for confounding risk factors are a weakness of studies on the influence of physical activity on the development of RA. A healthy lifestyle in terms of diet, smoking and body mass index can be assumed in participants with increased physical activity. An independent evaluation of the effect of physical activity on the development of RA does not seem feasible with the observational analyses conducted to date.

## 9. Conclusions

Environmental factors influence the development and the course of rheumatoid arthritis. Many environmental factors interact directly with predisposing genetic risk factors. Such a relationship has been described, for example, for the interaction between smoking and SE in the development of ACPA [73,74,78]. Some environmental factors such as smoking, dietary intake or physical activity could be influenced by the patient. The knowledge of the impact of individual lifestyle components on the progression or development of the disease enables doctors to provide adequate counselling for patients or those at risk.

The extent to which a single factor contributes to the development of the disease is often difficult to assess. Many risk factors occur in clusters. This holds true for dietary habits, nicotine consumption, physical activity and socioeconomic status—factors that all depend on educational status. Estimating their individual effects requires large sample sizes and demanding stratification procedures. Furthermore, the onset of rheumatoid arthritis itself may influence the lifestyle and socioeconomic status of patients. These reciprocal effects have to be taken into account as well.

The identification of lifestyle factors connected to the origin and course of RA relies on the retrospective analysis of large cohorts, although in some of these epidemiological studies, the data were gathered prospectively. Therefore, a causal influence of individual environmental factors on RA is difficult to prove. Nevertheless, the evidence for the influence of smoking on the development and an unfavorable course of RA is convincing. For other environmental factors, the evidence is not as strong. It can be assumed with confidence, that a healthy diet, as described above, frequent physical activity and a high socioeconomic status are associated with a more favorable course of the disease. To some extent, these factors also protect from the development of RA. However, the magnitude of influence and the relationship between the individual factors should be defined more precisely in future investigations.

## Data Availability

Not applicable.

## Supplementary Materials

The following supporting information can be downloaded at: https://www.mdpi.com/article/10.3390/jcm11237179/s1, Table S1: Studies investigating the impact of nutrition.

## Abbreviations

ACPA	anti-citrulline antibody
ACR20	American College of Rheumatology response criteria
ADIRA	anti-inflammatory diet in RA
AHEI 2010	Alternative Healthy Eating Index 2010
BMI	body mass index
CCP	cyclic citrullinated peptide
CRP	C-reactive protein
DAS28	Disease Activity Score
DMARD	Disease-modifying antirheumatic drug
EDII	Empirical Dietary Inflammatory Index
EIRA	Epidemiological Investigation of RA
ESR	erythrocyte sedimentation rate
EULAR	European League Against Rheumatism
HAQ	Health Assessment Questionnaire
HLIS	Healthy Lifestyle Index Score
HR	hazard ratio
ILD	interstitial lung disease
IRD	inflammatory rheumatic disease
MD	Mediterranean diet
NET	neutrophil extracellular trap
NHS	Nurses’ Health Study
NIEHS	National Institute of Environmental Health Sciences
NSAID	non-steroidal anti-inflammatory drug
OR	odds ratio
PUFA	polyunsaturated fatty acid
RA	rheumatoid arthritis
RF	rheumatoid factor
RR	relative risk
SE	shared epitope
