# Supplementary material for: Lifestyle Factors and Their Influence on Rheumatoid Arthritis: A Narrative Review
Source: J Clin Med. 2022 Dec 2;11(23):7179. doi: 10.3390/jcm11237179 (PMC9736780; doi:10.3390/jcm11237179)
Supplement: Supplementary file 1 [file jcm-11-07179-s001.zip › jcm-1984478-supplementary.pdf]

Journal of
Clinical Medicine

MDPI

Table S1: Studies investigating the impact of nutrition

Author Year Country	Patients (n)	Design	Population	Exposure	Outcome
Gan et al., 2017, [8], USA	67 cases 60 controls	case-control	at risk for RA	omega-3 fatty acid	RF positivity
Parks et al., 2013, [9], USA	424 cases 50,460 controls	case-control	RA/Non-RA	socioeconomic factors	development of RA
Johansson et al., 2018, [15], Sweden	1,721 cases 3,667 controls	case-control	RA/Non-RA	MD	development of RA
Nguyen et al., 2022, [17], France	480 cases 62,149 controls	case-control	RA/Non-RA	fish	development of RA
De Pablo et al., 2018, [21], Italy and Spain	96 cases 258 controls	case-control	RA/Non-RA	PUFA	development of RA
Bergström et al., 2013, [28], Sweden	172 cases 688 controls	case-control	RA/Non-RA	education, smoking, alcohol	development of RA
Ma et al., 2008, [7], USA	1,958	cross-sectional	Non-RA	dietary fiber	markers of systemic inflammation
Gan et al., 2017, [8], USA	2,166	cross-sectional	at risk for RA	omega-3 fatty acid	RF positivity
Tabung et al.; 2016, [11], USA	5,230	cross-sectional	Non-RA	NA	development of EDII
Tabung et al.; 2016, [11], USA	3,633	cross-sectional	Non-RA	diet	markers of systemic inflammation
Nguyen et al., 2022, [13], Korea	33,966	cross-sectional	Non-RA	nutrients, fruits, green vegetable	development of RA/ osteoarthritis
Ferguson et al., 2019, [25], UK	502,417	cross-sectional	Non-RA	central adiposity	development of RA
Hu et al., 2017, [10], USA	169,989	prospective cohort	Non-RA	diet	development of RA
Sparks et al., 2019, [12], USA	173,560	prospective cohort	Non-RA	diet	development of RA
Hu et al., 2015, [14], USA	174,638	prospective cohort	Non-RA	MD	development of RA
Pedersen et al., 2005, [18], Denmark	57,053	prospective cohort	Non-RA	diet	development of RA
Mazzuca et al., 2022, [19], UK	479,494	prospective cohort	Non-RA	diet	development of RA
Giuseppe et al., 2014, [20], Sweden	32,232	prospective cohort	Non-RA	PUFA	development of RA
Marchand et al., 2022, [22], USA	108,505	prospective cohort	Non-RA	weight gain	development of RA
Hair et al., 2013, [24], Netherlands	55	prospective cohort	at risk for RA	smoking, BMI	development of RA
Marchand et al., 2021, [26], USA	50,682	prospective cohort	Non-RA	waist circumference, BMI	development of RA

Bao et al., 2013, [37], USA	118,962	prospective cohort	Non-RA	nut consumption	mortality
Karlson et al., 2008, [67], USA	39,144	prospective cohort	Non-RA	vitamin E	development of RA
Sparks et al., 2015, [50], USA	53	retrospective cohort	RA	bariatric surgery	disease activity
Hafström et al. 2001, [34], Sweden	66	RCT	RA	vegan, gluten-free diet	pain
Lozada-Mellado et al., 2022, [36], Mexico	90	RCT	RA	exercise vs. MD	cytokines
Estruch et al., 2018, [38], Spain	7447	RCT	Non-RA	diets	mortality
Kjeldsen-Kragh et al., 1991, [41], Norway	53	RCT	RA	fasting and vegetarian diet	disease activity
Sköldstam et al., 2003, [42], Sweden	51	RCT	RA	MD vs. control diet	DAS28
Vadell et al., 2020, [44], Sweden	50	RCT	RA	intervention diet vs. control diet	DAS28
Laar et al., 1992, [47], Netherlands	94	RCT	RA	allergen-free vs. allergen-restricted diet	disease activity
Guagnano et al., 2021, [48], Italy	40	RCT	RA	exclusion diet vs. balanced diet	disease activity
Ranganath et al., 2022, [51], USA	40	RCT	RA	diet intervention	adiponectin, leptin, DAS28, HAQ
Alipour et al., 2014, [55], Iran	46	RCT	RA	probiotics	cytokines, DAS28
Hattaka et al., 2003, [56], Finland	21	RCT	RA	probiotics	cytokines, SJC, TJC
Pineda et al., 2011, [57], Canada	29	RCT	RA	probiotics	cytokines, ACR20, HAQ
Remans et al., 2004, [65], Netherlands	66	RCT	RA	PUFA	TJC
Di Giuseppe et al., 2014, [16], multi-national	174,701	meta-analysis	RA/Non-RA	fish	development of RA
Qin et al., 2015, [23], multi-national	11 studies	meta-analysis	RA/Non-RA	BMI	development of RA
Jin et al.,2013, [29], multi-national	195,029	meta-analysis	RA/Non-RA	alcohol consumption	development of RA
Schönenberger et al., 2021,	7 RCTs	meta-analysis	RA	diets	pain

[33], multi-national					
Giosuè et al., 2022, [39], multi-national]	19 studies	meta-analysis	Non-RA	fatty fish vs.lean fish	mortality, cardiovascular events
Müller et al., 2001, [46], multi-national	4 RCTs	meta-analysis	RA	fasting followed by vegetarian diet	pain
Aqaeinezhad Rdudbane et al., 2018, [54], multi-national	153<br>4 RCTs	meta-analysis	RA	probiotics	cytokines, DAS28
Goldberg et al., 2007, [59], multi-national	17 RCTs	meta-analysis	RA	omega-3 fatty acid	pain
Miller et al., 2005, [68], multi-national	139,967	meta-analysis	Non-RA	high-dosage vitamin E	mortality
Kou et al., 2022, [69], multi-national	39,845	meta-analysis	RA	vitamin E	TJC, SJC

ACR20, American College of Rheumatology response criteria; BMI, body mass index; DAS28, disease activity score; EDII, Empirical Dietary Inflammatory Index; HAQ, health assessment questionnaire; NA, not applicable; PUFA, polyunsaturated fatty acids; RA, rheumatoid arthritis; RCT, randomised controlled trial; RF, rheumatoid factor; SJC, swollen joint count; TJC, tender joint count.
